# Polarization-encoded color images for information encryption enabled by HfN refractory plasmonic metasurfaces

**DOI:** 10.1515/nanoph-2025-0502

**Published:** 2025-11-10

**Authors:** Yu-Cheng Chu, Tzu-Yu Peng, Chen-Yu Wang, Shyr-Shyan Yeh, Jia-Wern Chen, Yu-Jung Lu

**Affiliations:** Research Center for Applied Sciences, 38017 Academia Sinica, Taipei, 11529, Taiwan; Department of Physics, National Taiwan University, Taipei, 10617, Taiwan

**Keywords:** refractory plasmonics, polarization-encoded, image encryption, plasmonic colors, transition metal nitrides, metasurfaces

## Abstract

Polarization control plasmonic nanostructures provide a unique route to manipulate light–matter interactions at the nanoscale and are particularly powerful for information security applications, where polarization-encoded color images can be used for optical encryption and anticounterfeiting. Conventional plasmonic materials such as Au and Ag, however, suffer from poor thermal stability, limiting their integration into robust, CMOS-compatible devices. Here, we present a polarization-encoded color image platform based on refractory HfN plasmonic metasurfaces, which combine gold-like optical properties with exceptional hardness, compositional tunability, and superior high-temperature resilience. Periodically patterned HfN nanoantennas with widths of 200 nm exhibit well-defined localized surface plasmon resonances in the visible spectrum (628 and 564 nm) and can be selectively excited by orthogonal linear polarizations. We designed and realized a polarization-encoded color image in which distinct color channels are revealed under x- and y-polarized illumination, enabling decryption of hidden information. Under unpolarized illumination, the superposition of color channels effectively conceals the message, achieving robust optical encryption. Our results establish HfN plasmonic nanostructures as a key material platform for next-generation nanophotonics, uniquely combining gold-like optical properties with exceptional thermal robustness. Even after high-temperature annealing, HfN retains its plasmonic response, enabling reliable polarization-resolved color image encoding and decryption. This breakthrough paves the way for thermally resilient metasurfaces for secure data encryption, anticounterfeiting, and robust operation in extreme environments.

## 1 Introduction

Polarization control has emerged as a powerful degree of freedom for manipulating light at the nanoscale through various structural designs [1], [2], [3], [4], [5], [6], [7], [8], [9], [10], [11], [12], enabling applications ranging from color routing and beam shaping to high-density optical data storage [7], [9], [11], anticounterfeiting [8], [10], and secure information encryption [2], [3], [4], [11], [13]. Unlike amplitude or phase modulation, polarization-encoded information offers an additional channel for multiplexing, allowing multiple images or color patterns to be selectively retrieved under specific polarization states. In particular, polarization-encoded color images have recently attracted great interest as they enable intuitive, high-contrast readout and robust optical security features that are invisible under unpolarized light. Plasmonic nanostructures provide an ideal platform for realizing polarization-resolved optical functionalities, owing to their ability to concentrate electromagnetic fields into deep-subwavelength volumes and generate well-defined localized surface plasmon resonances (LSPRs) [14], [15], [16], [17]. Plasmonics have diversified optoelectronic characteristics [18], [19], [20], [21], including light focusing [18], plasmonic color generation [22], [23], strong localized electric fields [24], [25], [26], wavefront engineering [27], and wave guiding [28]. Based on the aforementioned properties, plasmonics has been widely applied in nanolasers [29], [30], [31], [32], [33], photodetection [34], [35], [36], [37], [38], photovoltaics [39], [40], and biochemical sensing [28], [41], [42]. By tailoring the geometry and orientation of plasmonic antennas, the spectral response can be designed to respond selectively to a given polarization state, enabling color-selective image reconstruction and optical encryption schemes. Previous demonstrations using Au, Ag, and Al nanoantennas have shown polarization-multiplexed holography and full-color generation [13], [24], [43], [44], [45], [46], [47]; however, noble metals suffer from low melting points, poor mechanical hardness, and chemical instability at the nanoscale [48], [49], [50], [51], [52], [53], [54], which limit their integration into robust and thermally resilient photonic systems. To overcome these challenges, transition metal nitrides (TMNs) such as TiN, ZrN, and HfN have emerged as CMOS-compatible plasmonic materials that combine gold-like optical properties with exceptional hardness, tunable stoichiometry, and high melting points (>3000 K) [48], [49], [50], [51], [52], [53], [54], [55], [56]. Notably, TMN-based plasmonic devices exhibit superior chemical stability compared to their noble-metal counterparts [49], [50], [57]. Among the TMNs, HfN stands out due to its relatively high bulk plasmon frequency (*λ*∼ 400 nm) and large negative real permittivity, which enable intense local electromagnetic field confinement and support localized surface plasmon resonance (LSPR) in the visible region. In this work, we focus on refractory HfN, characterized by its high bulk plasmon frequency (∼3.1 eV) and excellent thermal stability, which together facilitate visible-range LSPR, making it well suited for color imaging applications and highly compatible with semiconductor backend processes [37], [51]. Importantly, HfN nanostructures maintain their optical performance even after annealing at 900 °C, enabling reliable polarization-encoded color images that remain functional under harsh thermal environments, an essential requirement for practical data encryption and anticounterfeiting applications.

In this work, we optimize the growth of plasmonic HfN thin films, characterize their optical constants and crystallinity, and fabricate polarization-sensitive HfN plasmonic metasurfaces (PM) with precisely tuned dimensions. By orthogonally arranging two sets of antennas with distinct LSPR wavelengths, we realize a polarization-encoded QR code that selectively reveals distinct color channels under *x*- and *y*-polarized illumination, but remains concealed under unpolarized light. This approach establishes HfN PM as a robust, thermally stable platform for next-generation polarization-resolved nanophotonic devices and secure optical information encoding.

## 2 Experimental section

### 2.1 Fabrication of HfN plasmonic metasurfaces

HfN PM, consisting of patch antennas with an HfN back reflector, were fabricated using radio-frequency (RF) sputtering and electron-beam lithography (EBL). Before HfN deposition, the substrates were ultrasonically cleaned in acetone, isopropanol, and deionized water, successively, for 10 min per solvent. Afterward, the 105-nm-thick HfN thin films were deposited on the cleaned substrates in an RF sputter system with varied substrate temperature from RT to 800 °C, Ar flow rate 12 sccm, growth pressure three mTorr, and RF power 80 W. Regarding the EBL process, the electron photoresist ZEP-520A was spin-coated onto HfN thin films under the following conditions: 500 rpm for 5 s and 4,000 rpm for 1 min, in sequence. After the sample was baked at 180 °C for 3 min, the photoresist-covered HfN thin films were placed inside the electron beam writer (ELS-7000, ELIONIX INC.) to define the patch patterns. The dose of the electron beam is 0.15 μs/dot. Once the patch patterns were defined, a 40-nm-thick Cr layer was deposited on the patterns to serve as a hard mask. The residual electron photoresist and the Cr on it were lifted off by N, N-dimethylacetamide. Afterward, the non-Cr-covered HfN were etched through inductively coupled plasma reactive ion etching (Plasmalab System 100, Oxford Instruments) in the condition of Cl_2_ 8 sccm, Ar 8 sccm, growth pressure 8 mTorr, RF power 30 W, and ICP power 400 W. The substrate temperature was controlled at 20 °C. Finally, the samples were dipped into the Cr etchant to remove the residual Cr mask, completing the entire device fabrication process. The process flow is shown in Figure S1 of the supplementary information.

### 2.2 Optical characterization

Spectroscopic ellipsometry (VASE, J.A. Woollam Co.) combined with Drude–Lorentz model fitting was used to extract the complex permittivity and determine the optimal growth parameters for HfN growth. The crystalline quality and chemical composition of the HfN films were characterized by X-ray diffraction (XRD) and X-ray photoelectron spectroscopy (XPS), respectively. Atomic force microscopy (AFM; Asylum Research, Oxford Instruments) was used to assess surface roughness. The overall morphology of the HfN patch antennas was examined by scanning electron microscopy (SEM; FEI). The optical images were acquired using an optical microscope (BX51M, OLYMPUS, Japan). A high-extinction-ratio linear polarizer (extinction ratio ∼10,000:1) was employed to control the incident polarization state. The optical images and QR code demonstrations were recorded in a normal-incidence reflection geometry using a 10 × objective lens with a numerical aperture (NA) of 0.3. A broadband xenon lamp served as the illumination source, providing uniform and stable illumination throughout the measurements. The reflectance spectra of the patch antenna were obtained using a custom-built system integrating the microscope, light source, and a spectrometer. The area of interest was defined by a field aperture and confirmed using a CCD camera. The reflected light was collected by the spectrometer, covering a spectral range of 400–900 nm. The optical response was calibrated against a 40-nm-thick Ag film on an MgO substrate prior to measurement. All optical measurements were carried out in ambient air at room temperature and atmospheric pressure.

### 2.3 Numerical simulations

The reflectance spectra and electric field distributions were simulated using the finite-difference time-domain (FDTD) method. The permittivity of the HfN thin films was extracted from ellipsometry measurements and fitted using a Drude–Lorentz model. In the simulation, a broadband plane wave was incident on a single unit cell of the HfN patch antenna array, with periodic boundary conditions applied along the in-plane (*x*–*y*) directions and perfectly matched layers (PML) along the excitation (z) direction. A nonuniform mesh with a minimum size of 5 nm was used to resolve the HfN patch antennas and the HfN back reflector regions.

## 3 Results and discussion

**Figure 1:**
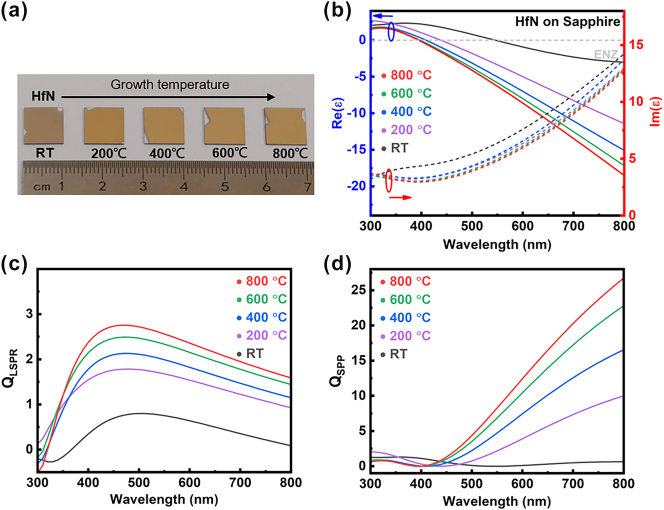
Optical properties of plasmonic HfN films grown at different temperatures on sapphire. (a) Growth of HfN films on sapphire substrates at temperatures from room temperature (RT) to 800 °C. (b) Real and imaginary parts of the dielectric function of HfN films deposited at temperatures ranging from RT to 800 °C. The epsilon-near-zero (ENZ) wavelength blue-shifts with increasing growth temperature, indicating tunable optical properties. The film deposited at 800 °C exhibits an ENZ wavelength shorter than 400 nm. (c) Calculated quality factor of the localized surface plasmon resonance (Q_LSPR_) and (d) calculated quality factor of the surface plasmon polariton (Q_SPP_) derived from the measured dielectric functions. The 800 °C film shows the highest Q_LSPR_ and Q_SPP_ across the visible and near-infrared ranges.

Since the substrate strongly influences the quality of deposited films, we first investigated how different substrate types affect the optical properties of HfN thin films. HfN films were reactively sputtered on MgO(100), c-plane sapphire(0001), Si, and SiC under the deposition conditions summarized in Table S1, followed by variable-angle ellipsometry measurements performed from 65° to 75°. The dielectric functions were extracted using the Drude–Lorentz model fitting over the wavelength range of 300–800 nm, as shown in Figure S2. Detailed fitting parameters are provided in Tables S2 and S3. In particular, we focus on HfN films grown on sapphire substrates because of their superior compatibility with CMOS fabrication processes. Notably, the films deposited on sapphire exhibit the lowest imaginary part of the dielectric function, which indicates reduced optical losses compared with those on other substrates. This can be attributed to their better crystallinity, which effectively suppresses electron scattering. These results highlight sapphire as a promising substrate for achieving low-loss plasmonic HfN films. Photographs of HfN films grown on sapphire substrates at different deposition temperatures (RT, 200 °C, 400 °C, 600 °C, and 800 °C). The film color progressively changes with increasing growth temperature, indicating variations in crystallinity and optical properties, as shown in Figure 1a. The dielectric functions of these films exhibit a clear and well-defined ENZ crossing, as shown in Figure 1b. The real part of the dielectric function, Re(ε), decreases monotonically with increasing wavelength for all deposition temperatures, with a distinct zero-crossing point corresponding to the epsilon-near-zero (ENZ) wavelength. As the growth temperature increases from room temperature (RT) to 800 °C, the entire Re(ε) curve shifts downward, resulting in a progressively shorter ENZ wavelength. In particular, the film grown at 800 °C shows an ENZ point at a wavelength shorter than 400 nm, indicating that higher-temperature deposition leads to more optically metallic behavior and improved plasmonic response in the visible range.

In addition, we found that the ENZ wavelength of HfN varies depending on the substrate, resulting in distinct plasmonic properties (Figure S2). Specifically, when the growth temperature is fixed at 800 °C, the ENZ wavelengths of HfN grown on MgO, sapphire, Si, and SiC are 364, 392, 365, and 366 nm, respectively. The imaginary part of the dielectric function Im(ε), which represents optical losses, generally decreases with increasing growth temperature across the visible spectrum. Films deposited at higher temperatures (600 °C and 800 °C) exhibit notably lower Im(ε) values, indicating reduced dissipative loss and improved plasmonic performance. In contrast, the room-temperature (RT) film shows the highest Im(ε), suggesting higher optical damping. The systematic reduction of Im(ε) with growth temperature highlights that high-temperature deposition yields better metallic HfN films with fewer defects and lower absorption losses, which is advantageous for plasmonic resonances. The improvement is expected to saturate beyond a certain temperature, as the reduction of extrinsic losses reaches a limit once the film approaches its optimal crystalline quality. In this work, growth at 800 °C already yields well-crystallized films with low optical loss. To evaluate the plasmonic performance of these films, we calculated the quality factor of the localized surface plasmon resonance (Q_LSPR_) and surface plasmon resonances (Q_SPP_) using Eq. (1) and Eq. (2) [52], [58]:

(1) $$Q_{\text{LSPR}}=\frac{1}{2}\frac{\omega }{\text{Im}\;\left(\varepsilon \right)}\frac{d}{d\omega }\text{Re}\;\left(\varepsilon \right)$$

(2) $$Q_{\text{SPP}}=\text{Re}\;{\left(\varepsilon \right)}^{2}/\text{Im}\;\left(\varepsilon \right)$$

where ω represents the angular frequency, and Re(ε) and Im(ε) denote the real and imaginary parts of the dielectric function ε, respectively. Figure 1c shows the calculated Q_LSPR_ for HfN films deposited at different temperatures. Q_LSPR_ exhibits a distinct peak in the visible spectral range, with both the peak value and overall magnitude increasing systematically with growth temperature. The film deposited at 800 °C shows the highest Q_LSPR_ (∼2.5) across the entire spectrum, indicating the lowest plasmon damping and strongest resonance quality. In contrast, the RT film displays a significantly lower Q_LSPR_, reflecting higher optical losses. These results highlight again that higher deposition temperatures yield HfN films with improved crystalline quality and reduced defect-induced scattering, thereby enhancing localized plasmonic performance. The calculated Q_SPP_ for HfN films deposited at different temperatures is shown in Figure 1d. Similar to Q_LSPR_, Q_SPP_ increases systematically with deposition temperature, with the 800 °C film showing the highest Q_SPP_ over the entire visible and near-infrared range. These results confirm that high-temperature growth significantly enhances the SPP quality, which is beneficial for applications requiring long-range plasmon propagation, such as waveguiding and on-chip photonic circuitry.

**Figure 2:**
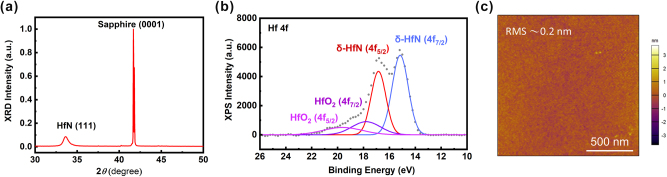
Structural and surface characterization of HfN films grown on sapphire. (a) XRD spectrum showing the (111) peak of HfN, confirming the film with a preferred (111) orientation. (b) XPS spectrum fitted with components corresponding to HfN and a minor HfO_2_ contribution, confirming the film stoichiometry. (c) AFM topography image showing an atomically smooth surface with an RMS roughness of approximately 0.2 nm. Scale bar: 500 nm.

It has been reported that both the stoichiometry and crystallinity of HfN play a critical role in determining its optical and electrical properties [51], [59], [60], [61]. For example, HfN is highly conductive [51], whereas *c*-Hf_3_N_4_ is nearly insulating [61]. To ensure that the HfN films grown on sapphire possess the desired metallic and crystalline quality for plasmonic applications, we carried out a comprehensive structural and compositional characterization. The XRD pattern (Figure 2a) reveals prominent reflections corresponding to the (111) planes of HfN (33.75°), indicating a preferred (111) orientation [62]. This preferred orientation is beneficial for achieving a uniform optical response. The measured peak widths (FWHM 0.58°) suggest good crystallinity, which is expected to reduce electron scattering and thereby minimize optical losses, as shown in Figure S2. XPS analysis further confirms that the film is predominantly metallic HfN, with only a minor contribution from surface-oxidized HfO_2_. Regarding the XPS spectrum of HfN/Sapphire shown in Figure 2b, the fitting curve shows four distinct peaks at 15.0 eV, 16.7 eV, 18.3 eV, and 19.8 eV. The first two peaks correspond to HfN (4f_7/2_) and HfN (4f_5/2_), respectively [59], [60], while the latter two are assigned to the HfO_2_ (4f_7/2_) and HfO_2_(4f_5/2_), respectively [63]. The significantly higher intensity of the HfN peaks indicates that the film composition is dominated by metallic HfN, with only slight surface oxidation. Such stoichiometric control is crucial for tuning the ENZ wavelength and optimizing the plasmonic performance. Finally, AFM image (Figure 2c) shows an exceptionally smooth surface with an RMS roughness of ∼0.2 nm, which is highly advantageous for nanofabrication and minimizes additional scattering losses at the metal–dielectric interface. Together, these results confirm that HfN grown on sapphire provides a high-quality, CMOS-compatible plasmonic platform with low optical damping, making it ideal for integration into metasurfaces and patch antennas requiring reproducible and robust plasmonic resonances.

**Figure 3:**
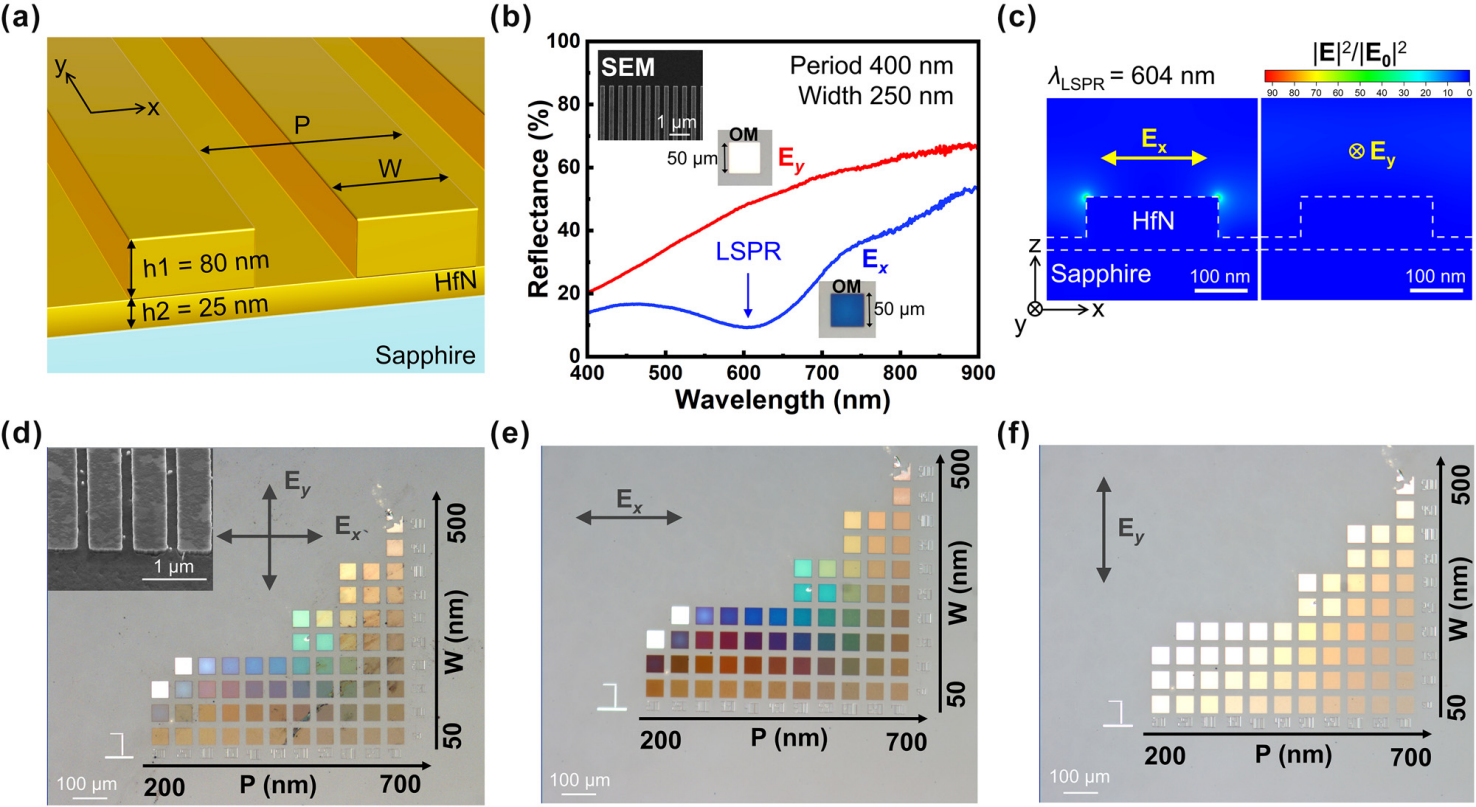
Design and polarization-dependent optical response of HfN plasmonic metasurfaces. (a) Schematic of the HfN PM consisting of patch antennas with an HfN back reflector on sapphire, showing the antenna width (W), period (P), and height (*h*_1_ = 80 nm, *h*_2_ = 25 nm). (b) Measured reflectance spectra under *x*- and *y*-polarized illumination, revealing a pronounced LSPR near 604 nm only for *E*_x_ excitation. Insets show SEM and optical microscope (OM) images of the fabricated polarization-sensitive plasmonic nanoantennas. The area of the color palette is 50 × 50 μm^2^. (c) Simulated electric-field intensity distribution for *x*- and *y*-polarized light at a resonant wavelength of 604 nm, confirming polarization-selective excitation of the LSPR mode. For a clearer comparison, the color scale bar of the field intensity plots was unified across both polarization states. (d) Optical microscope images of HfN polarization-sensitive PM with varying W and P under unpolarized illumination, revealing multiplexed color responses with low color purity. Insets show SEM images of the polarization-sensitive metasurfaces. (e,f) Polarization-resolved optical images under *x*- and *y*-polarized light illumination, respectively, where distinct color channels are selectively revealed, enabling polarization-controlled color imaging.

After confirming the crystallinity and stoichiometry of the HfN films, we fabricated polarization-sensitive plasmonic nanostructures based on the optimized growth conditions. The device design is shown in Figure 3a. Each metasurface consists of rectangular HfN patches with a long axis aligned along the *y*-direction, ensuring that the LSPR is selectively excited only under *x*-polarized (*E*_*x*_) light illumination. The patch height (*h*_1_) is 80 nm, while the patch width (W) and period (P) are tunable parameters that determine the LSPR wavelength and resulting reflected different colors. A thin HfN back reflector (*h*_2_ = 25 nm) underneath the patches ensures efficient light confinement and minimizes transmission losses. The measured polarization-dependent reflectance spectra in the spectral range from 400 nm to 900 nm for a representative device (*P* = 400 nm, *W* = 250 nm) are shown in Figure 3b. Under *E*_x_ illumination, a pronounced reflectance minimum appears near 604 nm, characteristic of LSPR absorption, whereas no distinct resonances are observed under *y*-polarized (*E*_y_) illumination, confirming the strong polarization selectivity. The reflectance minimum reaches 9.2 % at 604 nm, indicating strong light–matter coupling in the HfN nanostructure. The inset shows an SEM image of the patch antenna with a back reflector, while the OM images display the device under *E*_x_- and *E*_y_-polarized illumination. In addition, the simulated reflectance (Figure S3) also reveals similar polarization-sensitive behavior, but the reflectance minimum in the visible range is redshifted to 639 nm, and an additional reflectance minimum appears in the near-infrared (NIR) region. The slight spectral shift (∼35 nm) observed between the simulation and experiment is mainly attributed to fabrication imperfections and the presence of a thin surface oxide layer that was not considered in the initial simulation. The fabrication-induced discrepancies arise from unavoidable variations in the size, depth, and shape of the nanostructures during the lithography process. As confirmed by the XPS analysis (Figure 2b) and our previous work [57], a thin oxide layer may exist on the surface of the HfN films; however, its contribution is minor compared to the dominant metallic HfN signal. Based on the relative peak intensities in the XPS spectrum, the oxide thickness is estimated to be less than 3 nm. To assess its possible influence, we performed a sensitivity analysis using FDTD simulations by introducing a 3 nm conformal HfO_2_ overlayer on top of the designed nanostructure. The simulation shows that adding this surface oxide layer induces only a slight redshift of the resonant wavelength (approximately 30 nm) while exerting negligible influence on the resonance linewidth and contrast (Figure S4). Importantly, the presence of the surface oxide does not affect the polarization selectivity or the color-encoding functionality of the metasurface.

To understand the origin of the reflectance minimum, we simulated the electric field intensity distribution of the plasmonic nanostructure. FDTD simulations (Figure 3c) reveal a strongly localized electric field at the top corners of the HfN patches under *x*-polarized illumination, with a field enhancement exceeding 90 × relative to the incident field. In contrast, under *E*_y_ excitation, the field distribution is weak at the top corners of the HfN patches under *y*-polarized illumination | ***E*** |^2^/| ***E***_0_|^2^∼1, resulting in the absence of a pronounced resonance. A simple modal analysis indicates that the dominant contribution arises from a corner mode oriented along the *x*-axis. The anisotropy ratio of the corner electric field under *x*- and *y*-polarized excitation reaches approximately 9.5 at the resonance wavelength, highlighting the strong polarization selectivity of the mode. These results confirm that the reflectance minimum originates from a corner-localized LSPR mode dictated by the anisotropic patch geometry. Based on this concept, we fabricated HfN metasurfaces with periods ranging from 200 to 700 nm and widths ranging from 50 to 500 nm. Optical images under unpolarized and polarization-resolved illumination are shown in Figure 3d–f. Under unpolarized light, the color palette exhibits low-purity mixed colors (Figure 3d). The two nearly white areas are attributed to the fact that the nanostructures in these regions were not successfully fabricated during the nanofabrication process. As a result, these areas effectively behave as unpatterned thin films, exhibiting broadband reflection that appears white in color. In contrast, *E*_*x*_ illumination produces a vivid and tunable color response across the array (Figure 3e), while *E_γ_* illumination results in nearly uniform, pale reflection (Figure 3f), further confirming the polarization-encoded functionality. The vivid color response observed under *x*-polarized light illumination in Figure 3e is comparable to that reported by Chiao et al.; however, the reported devices did not exhibit polarization selectivity because they utilized disk-shaped nanostructures [51].

**Figure 4:**
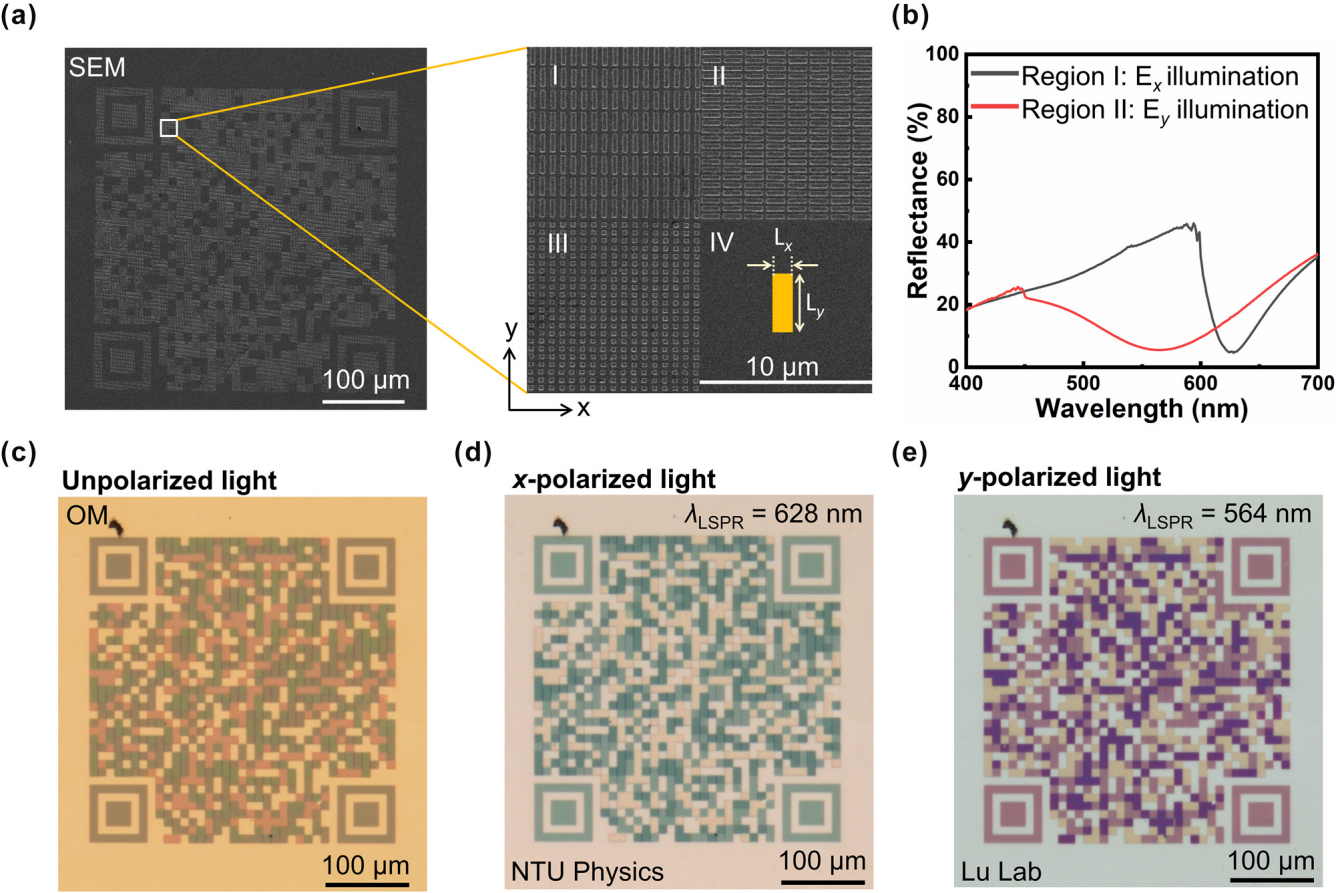
Polarization-encoded color image encryption with refractory HfN metasurfaces, retaining optical functionality after 900 °C annealing. (a) SEM image of the fabricated polarization-sensitive PM and magnified view showing orthogonally arranged HfN patch antenna metasurfaces in two regions with distinct dimensions (*L*_x_, *L*_y_). (b) Measured reflectance spectra of the two HfN PM regions under *x*- and *y*-polarized illumination, revealing distinct LSPR wavelengths at 628 nm and 564 nm. (c) OM image under unpolarized illumination showing a superimposed color pattern that conceals the encoded information. (d) Under *x*-polarized illumination, the first color channel is selectively revealed, displaying the QR code patterns directly to the website of NTU Physics. (e) Under *y*-polarized illumination, the second color channel is revealed, and the QR code patterns direct to the lab website. The polarization selectivity enables reliable decryption of encoded information and robust optical encryption.

Leveraging the polarization sensitivity of HfN PM, we demonstrate a proof-of-concept optical encryption platform based on polarization-encoded QR codes. As illustrated in Figure 4a, the metasurface is designed with four distinct regions consisting of orthogonally arranged HfN patch antennas (regions I and II), a square-patch array (region III) with *L*_*x*_ = *L*_*y*_ = 200 nm, and a planar HfN back reflector (region IV). For the vertical patch antenna with *P*_x_ = 600 nm, *L*_*x*_ = 200 nm, *L*_*y*_ = 1 μm, and *P*_*y*_ = 1.26 μm. Similarly, for the horizontal patch antenna with *P*_y_ = 450 nm, *L*_*y*_ = 200 nm, *L*_*x*_ = 1 μm, and *P*_*x*_ = 1.26 μm. The measured reflectance spectra (Figure 4b) confirm the polarization selectivity: region I exhibits a pronounced reflectance minimum at 628 nm under *E*_x_ illumination, while region II shows a distinct reflectance minimum at 564 nm under *E*_y_ illumination. Figure S5 displays the simulation results of polarization-controlled reflectance spectra. These spectrally separated resonances enable reliable channel multiplexing and minimize crosstalk between the two QR codes. The ability to selectively reveal independent information channels with orthogonal polarization states highlights the potential of this approach for secure optical data encoding and anti-counterfeiting applications. This orthogonal design ensures that under unpolarized illumination, the regions display mixed colors, concealing the embedded information (Figure 4c). When illuminated with *E*_x_ or *E*_y_ polarized light, only the corresponding subset of antennas is excited, revealing two independent QR code patterns (Figure 4d and e) that direct users to different websites. Notably, the entire metasurface retains its optical functionality even after annealing at 900 °C, highlighting the exceptional thermal stability of HfN compared to conventional noble-metal plasmonic materials. As demonstrated in our previous work, HfN plasmonic nanostructures exhibit no observable changes in resonance wavelength or morphology after high-temperature annealing [51]. Figure S6 shows the microscopy images of the HfN metasurfaces before annealing, revealing no noticeable changes in the image or morphology and confirming their excellent thermal robustness. This refractory nature ensures device reliability under extreme processing or operating conditions, making the platform suitable for integration into CMOS-compatible photonic circuits and high-temperature sensing environments. To assess the robustness of the polarization-encoded optical encryption, we performed additional measurements to evaluate the tolerance of the metasurface response to varying polarization angles. As shown in Figures S7 to S10, the polarization-encoded QR code image remains clearly distinguishable for polarization angle deviations of up to ±30° and ±40° from the nominal *x*- and *y*-polarization directions, respectively. Beyond this range, the color contrast gradually decreases, demonstrating good angular tolerance for practical operation. Together, these results demonstrate that HfN PM offer a robust, scalable, and polarization-programmable platform for next-generation optical encryption and secure information technologies.

## 4 Conclusions

In conclusion, we have demonstrated a robust and thermally stable polarization-sensitive plasmonic platform for optical encryption based on HfN PM that consists of patch antennas. Unlike conventional Au- or Ag-based plasmonic devices, which suffer from low melting points and chemical instability, HfN nanostructures preserve their crystallinity and plasmonic response even after high-temperature annealing (900 °C), ensuring reliable operation under harsh environments. The fabricated rectangular patch antennas exhibit well-defined LSPRs (628 nm and 564 nm) in the visible spectrum and strong polarization selectivity, enabling the construction of a polarization-encoded QR code with two orthogonal color channels. Under x- and y-polarized illumination, the encoded colors can be selectively decrypted; however, under unpolarized light, the superposition of the two channels conceals the information, thereby achieving a secure optical encryption scheme. This reliable polarization-encoded color image approach represents a significant advance for practical information security, anticounterfeiting, and high-temperature nanophotonic applications. By leveraging refractory HfN, we establish PM as an indispensable platform for polarization-encoded optical encryption, offering visible-color plasmonic resonances together with exceptional thermal stability and CMOS compatibility. This key material breakthrough enables robust and reproducible decryption of hidden information even under extreme thermal conditions, setting a new benchmark for secure, polarization-resolved nanophotonic devices and driving the next generation of optical information technologies.

## Supplementary Material

Supplementary Material Details
