# Supplementary material for: Polarization-encoded color images for information encryption enabled by HfN refractory plasmonic metasurfaces
Source: Nanophotonics. 2025 Nov 10;14(25):4655–64. doi: 10.1515/nanoph-2025-0502 (PMC12714051; doi:10.1515/nanoph-2025-0502)
Supplement: Supplementary file 1 — Supplementary Material Details [file j_nanoph-2025-0502_suppl_001.pdf]

Supporting Information for

Polarization-Encoded Color Images for Information Encryption Enabled by HfN Refractory Plasmonic Metasurfaces

Yu-Cheng Chu,[1,2] Tzu-Yu Peng,[1,2] Chen-Yu Wang,[1,2] Shyr-Shyan Yeh,[1,2] Jia-Wern Chen,[1] and Yu-Jung Lu[1,2]*

[1]*Research Center for Applied Sciences, Academia Sinica, Taipei 11529, Taiwan*
[2]*Department of Physics, National Taiwan University, Taipei 10617, Taiwan*

*To whom correspondence should be addressed.

*E-mail : yujunglu@gate.sinica.edu.tw

This PDF file includes:

Note S1. Device fabrication

Note S2. Characterization of HfN thin films

Note S3. Simulated reflectance spectra of HfN metasurfaces under *x*- and *y*- polarized light illumination

Note S4. Simulated reflectance spectra of region I and II under *x*- and *y*-polarized light illumination

Note S5. HfN plasmonic metasurfaces before annealing

Note S1. Device fabrication

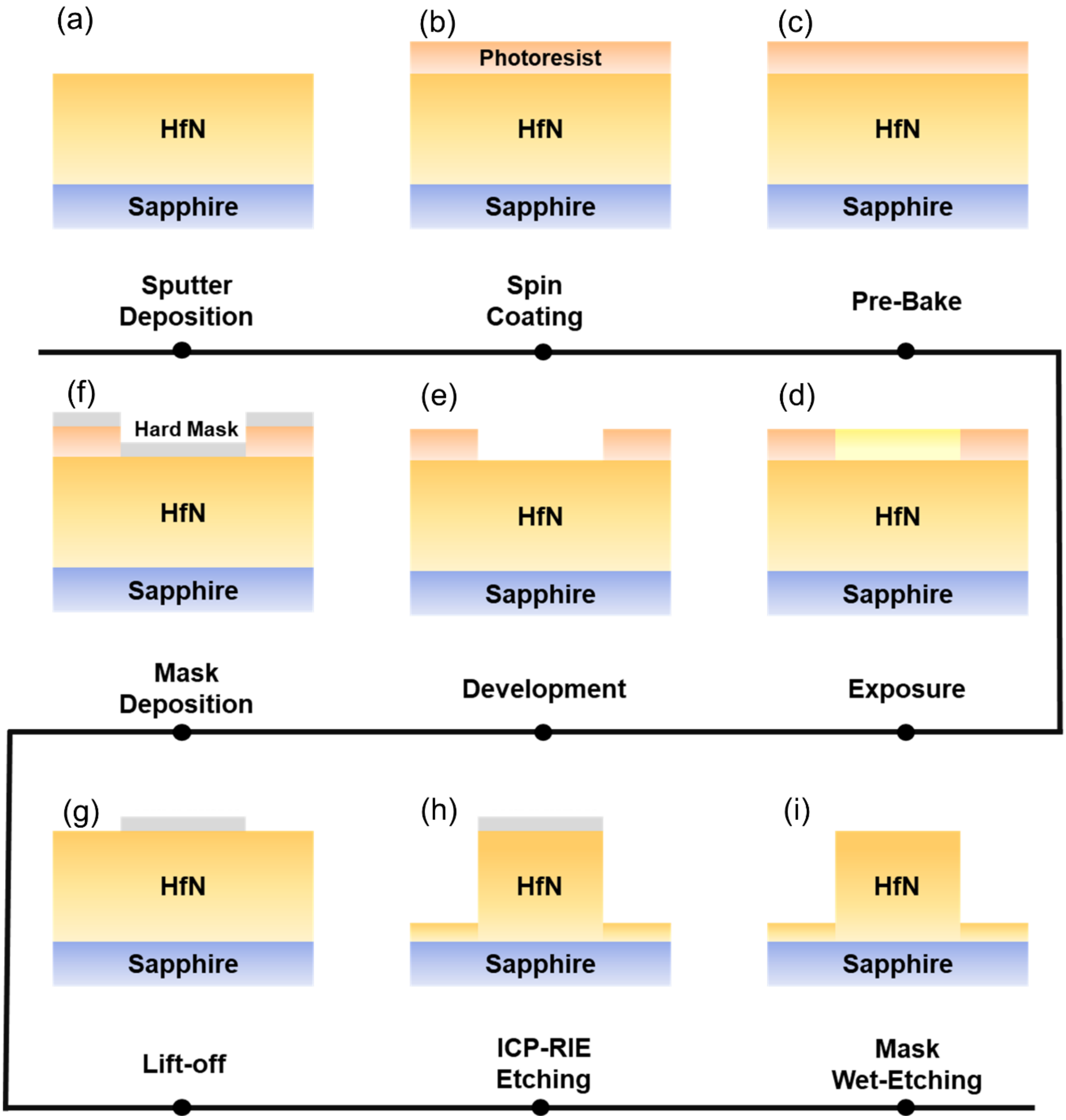

Figure S1. The fabrication process (side view) of the HfN patch antenna. **(a)** depositing HfN thin film on a sapphire substrate through sputtering. **(b)** spin-coating photoresist on as-prepared HfN thin film. **(c)** pre-baking photoresist before electron-beam exposure. **(d)** exposing the sample through a program-controlled electron beam. **(e)** developing the exposed area. **(f)** depositing Cr as the hard mask for the etching process through an electron-gun evaporator. **(g)** lifting off the residual photoresist and the Cr thereon. **(h)** etching the bare HfN through inductively coupled plasma reactive ion etching (ICP-RIE). **(i)** removing the Cr hard mask through Cr etchant to finish the HfN patch antenna fabrication process.

Note S2. Characterization of HfN thin films

Sample	HfN on MgO	HfN on sapphire	HfN on Si	HfN on 4H-SiC
**Target**	HfN	HfN	HfN	HfN
**Base pressure (Torr)**	1×10^{-8}	1×10^{-8}	1×10^{-8}	1×10^{-8}
**Growth pressure (mTorr)**	3	3	3	3
**Ar gas flow (sccm)**	12	12	12	12
**RF bias (V)**	360	360	360	360
**Growth temperature (°C)**	800	RT to 800	800	800
**Thickness (nm)**	105	105	105	105

Table S1. Growth parameters of the HfN on MgO, sapphire, Si, and 4H-SiC by RF sputtering.

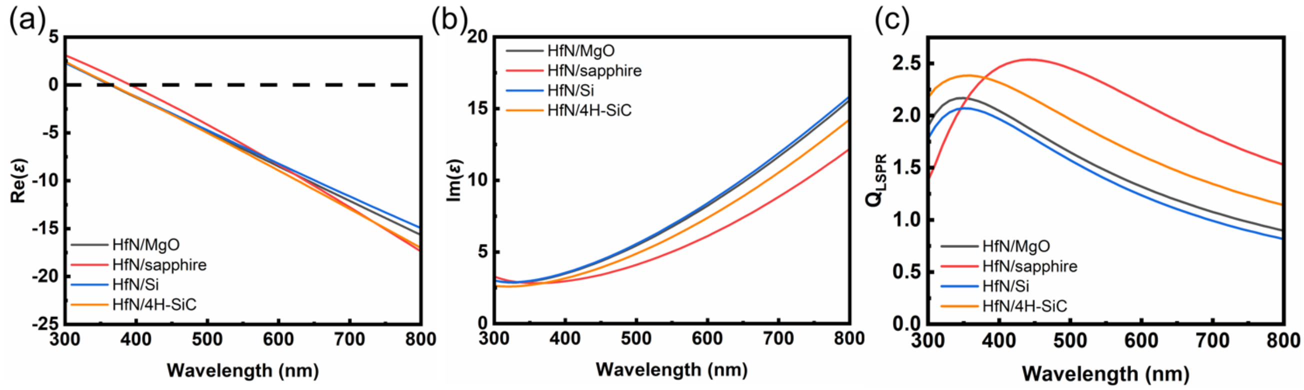

Figure S2. (a) real part and **(b)** imaginary part of permittivity of HfN thin films deposited on MgO, sapphire, Si, and 4H-SiC substrates. All the curves show the ENZ point at wavelengths shorter than 400 nm. The HfN film deposited on sapphire exhibits the lowest imaginary part of the dielectric function, which indicates reduced optical losses compared with those on other substrates. **(c)** The Q_{LSPR} derived from **(a)** and **(b).** The HfN deposited on sapphire has the highest Q_{LSPR} in the visible and near-infrared region.

The complex permittivity of HfN films can be described by the Drude-Lorentz model measured by an angle-dependent spectroscopic ellipsometer (VASE, J.A. Woollam Company)[1], which accounts for both intraband and interband transitions. The equation of the Drude-Lorentz model is shown in Eq. (S1)

$$\varepsilon = \mathrm{Re}(\varepsilon) + i\,\mathrm{Im}(\varepsilon) = \varepsilon_{\infty} - \frac{\omega_p^2}{\omega^2 + i\Gamma\omega} + \sum_{j=1}^{n} \frac{f_j \cdot \omega_{0j}^2}{\omega_{0j}^2 - \omega^2 - i\gamma_j\omega}, \qquad \text{Eq.(S1)}$$

Where complex permittivity ε is a function of the photon frequency, the first term ε_{∞} is the permittivity in the condition of ultra-high frequency. The second term represents the Drude model, i.e., the interaction between conduction electrons and electromagnetic waves. The parameter ω_p is the plasma frequency, and Γ is the collision rate. The third term relates to Lorentz oscillators, which account for the bound electrons contributing to optical absorption. The parameter f_j is the strength of the oscillator; ω_{oj} is the resonant angular frequency; and γ_j is the damping factor.

	High frequency permittivty	Drude term (intraband)		Lorentz oscillator 1 (interband)			Lorentz oscillator 2 (interband)			ENZ frequency	Fitting MSE
**Parameter**	ε_∞	ω_p (eV)	Γ (eV)	f_1 (eV)	ω_{o1} (eV)	γ_1 (eV)	f_2 (eV)	ω_{o2} (eV)	γ_2 (eV)	ω_s (eV)	MSE
**HfN on MgO**	3.38	7.05	0.63	14.43	1.02	2.15	5.87	5.56	1.81	3.38	1.38
**HfN on sapphire**	3.54	7.15	0.54	15.77	0.93	1.95	5.95	5.54	1.86	3.38	1.59
**HfN on Si**	3.58	6.95	0.66	14.50	1.04	2.19	5.51	5.36	1.65	3.38	1.05
**HfN on 4H-SiC**	3.12	7.20	0.56	14.36	0.95	2.03	6.74	5.77	1.89	3.38	0.99

Table S2. Ellipsometry fitting parameters for the complex permittivity of HfN thin films on MgO, sapphire, Si, and 4H-SiC. The Drude-Lorentz model consists of a Drude term and two Lorentz oscillators.

Growth temperature	High frequency permittivity	Drude term (intraband)		Lorentz oscillator 1 (interband)			Lorentz oscillator 2 (interband)			ENZ frequency	Fitting MSE
**T (°C)**	ε_∞	ω_p (eV)	Γ (eV)	f_1 (eV)	ω_{o1} (eV)	γ_1 (eV)	f_2 (eV)	ω_{o2} (eV)	γ_2 (eV)	ω_s (eV)	MSE
**RT**	5.07	4.04	1.06	18.62	1.10	1.50	2.60	3.86	2.69	2.28	12.66
**200**	4.31	5.23	0.62	30.24	0.83	1.18	3.07	4.98	3.30	2.82	7.01
**400**	4.89	6.05	0.56	31.08	0.81	1.18	2.83	4.37	2.37	3.02	6.46
**600**	4.91	6.40	0.48	33.22	0.76	1.13	2.80	4.45	2.32	3.10	6.82
**800**	5.25	6.16	0.53	34.16	0.81	1.02	2.79	4.11	1.83	3.14	7.40

Table S3. Ellipsometry fitting parameters for the complex permittivity of HfN thin films on sapphire under different growth temperatures (RT, 200 °C, 400 °C, 600 ˚C, and 800 ˚C). The Drude-Lorentz model consists of a Drude term and two Lorentz oscillators.

Note S3. Simulated reflectance spectra of HfN metasurfaces under *x*- and *y*- polarized light illumination

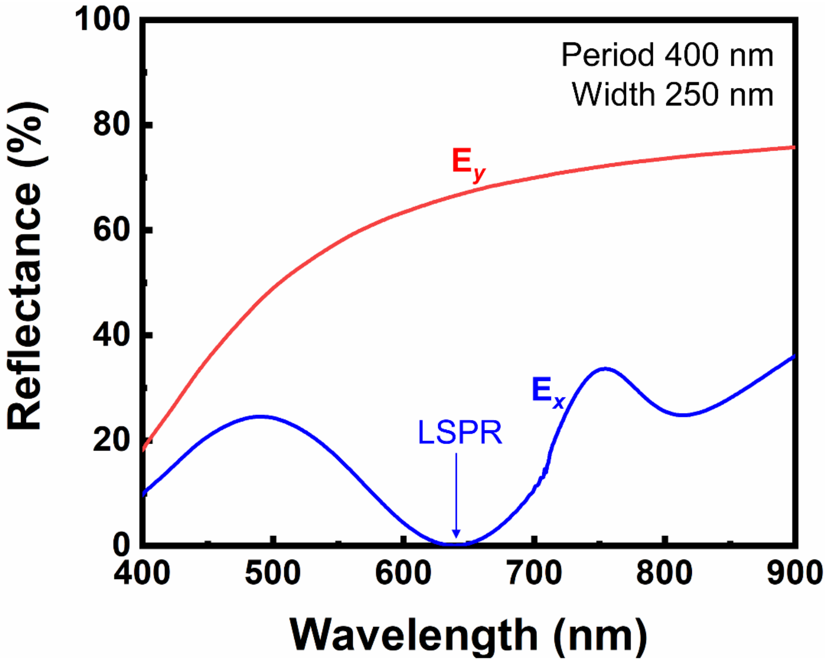

Figure S3. Simulated reflectance spectra of the HfN plasmonic metasurfaces with a period of 400 nm and a width of 250 nm under polarized light illumination (E_x and E_y).

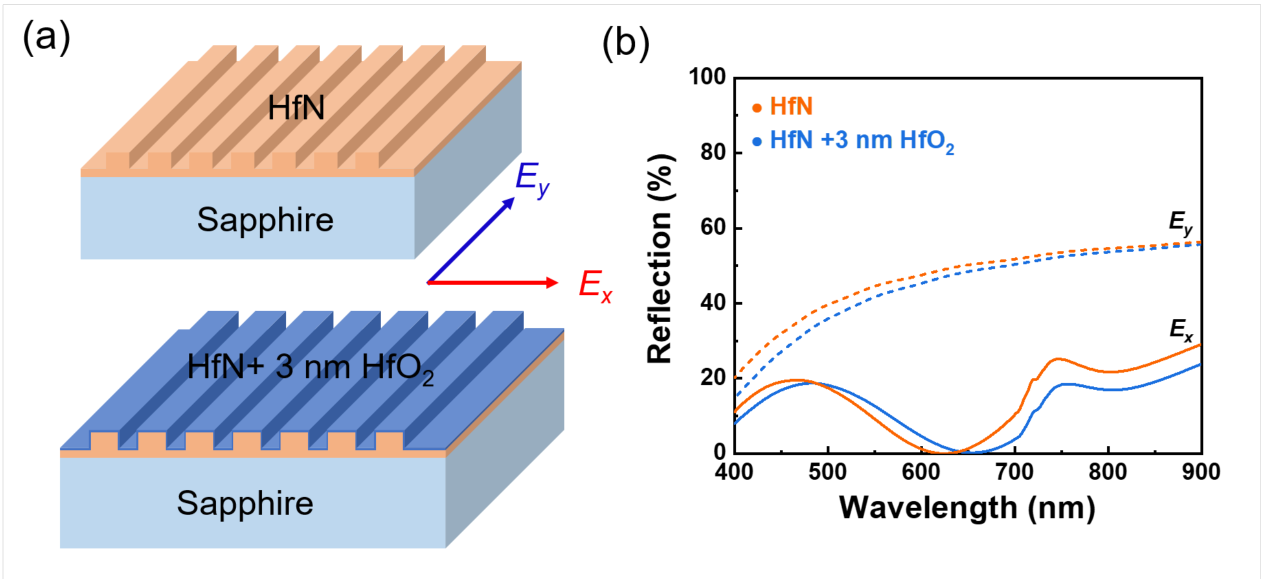

Figure S4. The simulation shows that adding a 3 nm surface oxide layer (HfO_2) on top of the designed nanostructure induces only a slight redshift of the LSPR peak (approximately 30 nm) and causes negligible changes in the resonance linewidth and contrast. The calculation results show that the presence of the surface oxide layer (HfO_2) does not affect the polarization selectivity.

Note S4. Simulated reflectance spectra of region I and II under *x*- and *y*-polarized light illumination

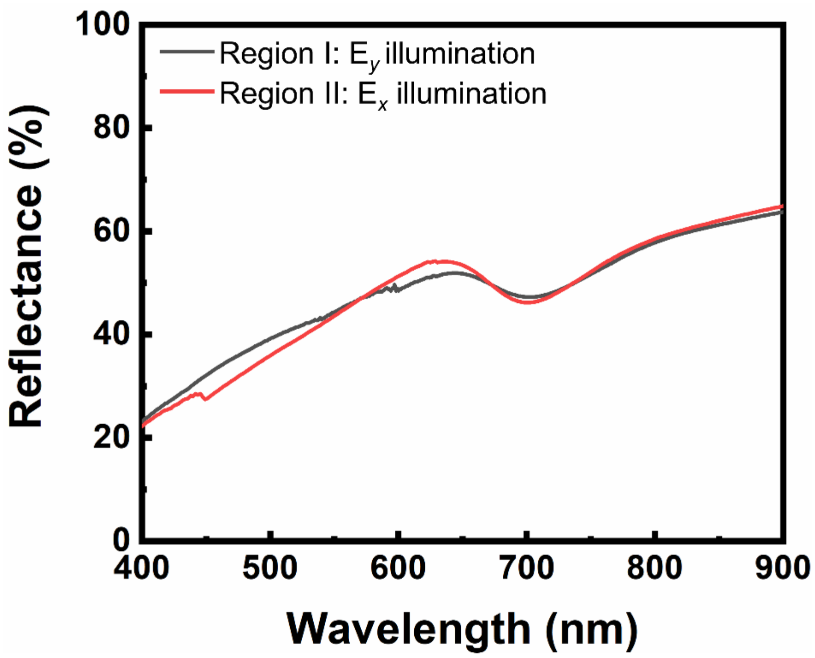

Figure S5. The simulated reflectance spectra of regions I and II under E_x and E_y illumination.

Note S5. HfN plasmonic metasurfaces before annealing

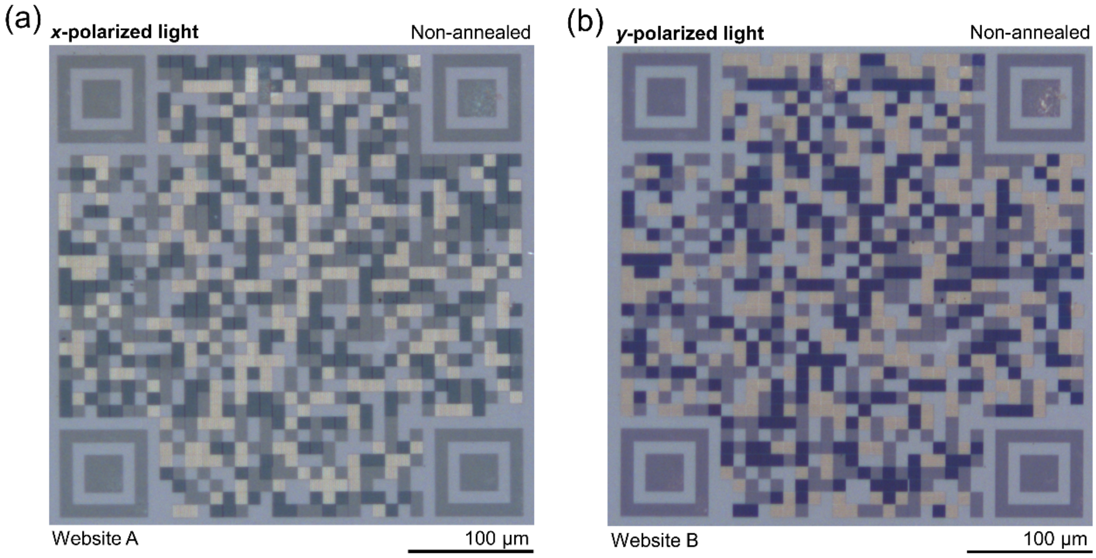

Figure S6. Polarization-encoded color image encryption using refractory HfN metasurfaces before annealing. The fabricated QR code patterns are shown under (**a**) *x*-polarized and (**b**) *y*-polarized light illumination. Both patterns direct users to the same websites as the QR codes measured after 900 °C annealing: pattern (**a**) links to website A, and pattern (**b**) links to website B.

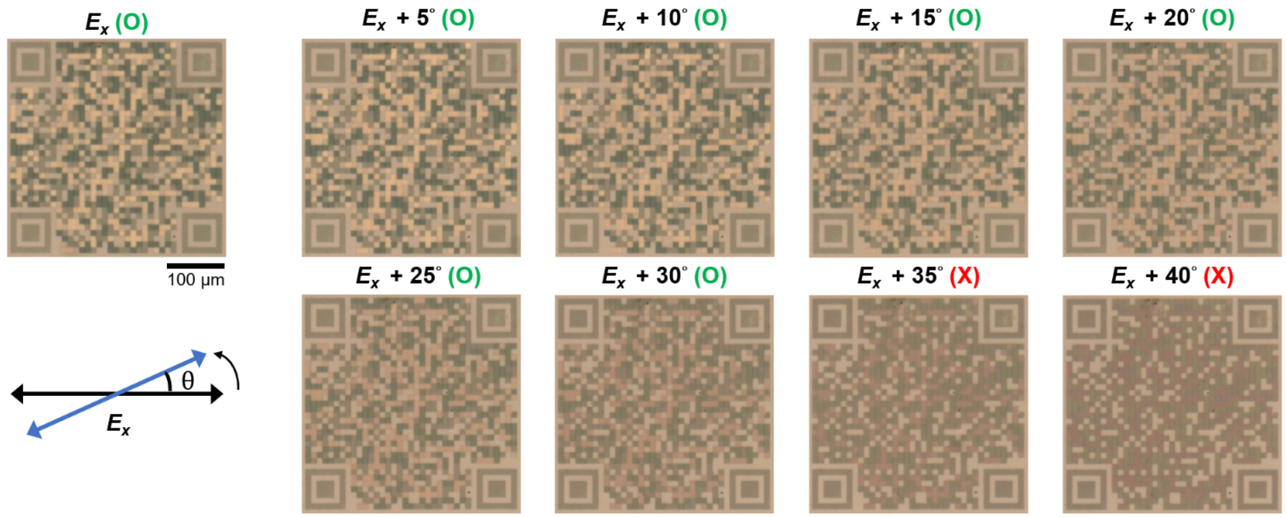

Figure S7. The encoded image remains clearly distinguishable for polarization angle deviations from 0° to 30° relative to the nominal *x*-polarization direction, and the QR code image links to website A. Beyond this range, the color contrast gradually decreases.

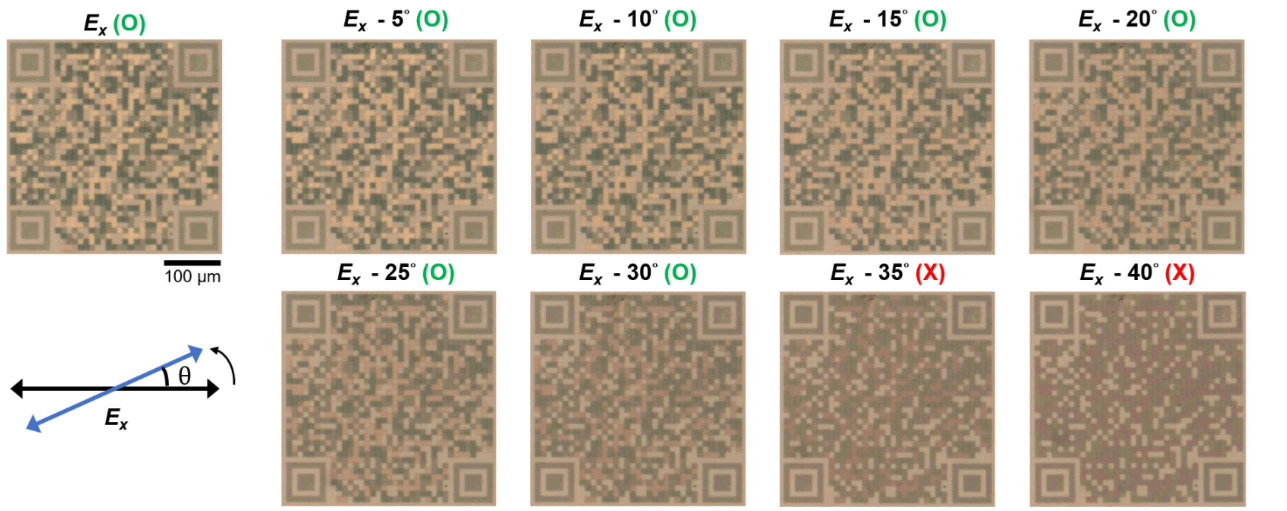

Figure S8. The encoded image remains clearly distinguishable for polarization angle deviations from 0° to –30° relative to the nominal *x*-polarization direction, , and the QR code image links to website A. Beyond this range, the color contrast gradually decreases.

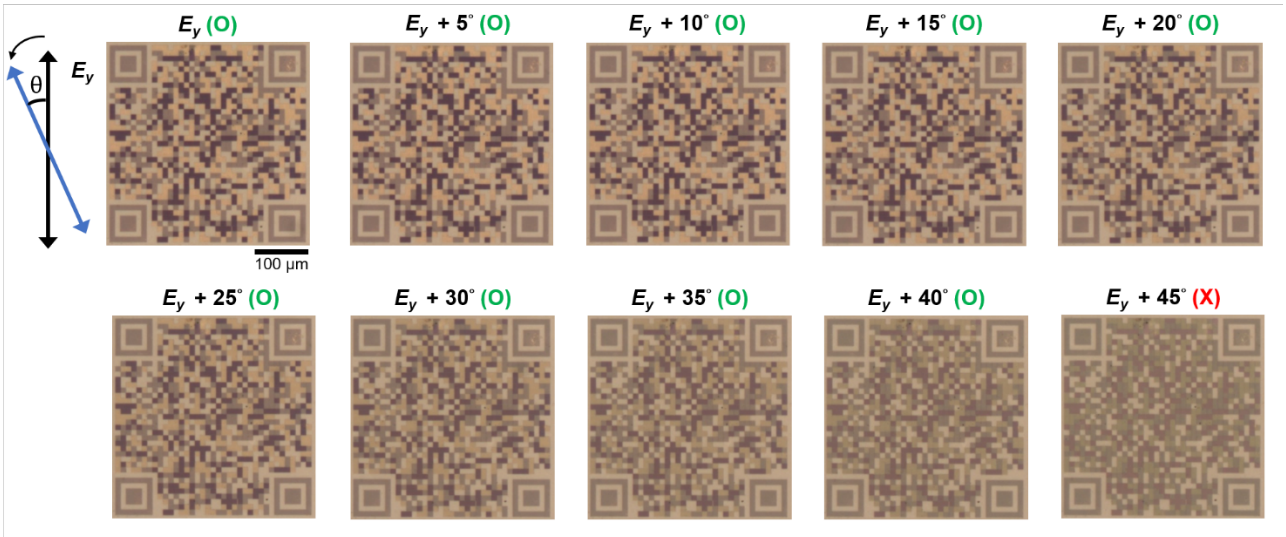

Figure S9. The encoded image remains clearly distinguishable for polarization angle deviations from 0° to 40° relative to the nominal *y*-polarization direction, and the QR code image links to website B. Beyond this range, the color contrast gradually decreases.

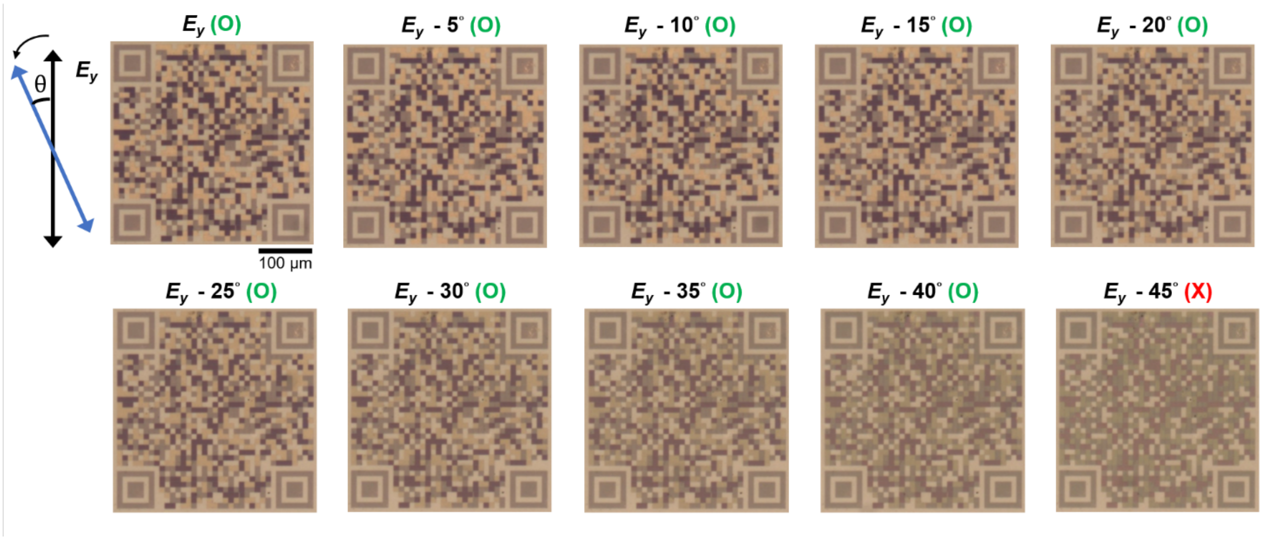

Figure S10. The encoded image remains clearly distinguishable for polarization angle deviations from 0° to –40° relative to the nominal *y*-polarization direction, and the QR code image links to website B. Beyond this range, the color contrast gradually decreases.

Reference:

[1]. J. N. Hilfiker, N. Singh, T. Tiwald, D. Convey, S. M. Smith, J. H. Baker, and H. G. Tompkins, "Survey of methods to characterize thin absorbing films with Spectroscopic Ellipsometry," *Thin Solid Films, vol.* 516, pp. 7979-7989, 2008.
